# Primary level management of eye injury/trauma

**Published:** 2013

**Table:** 

	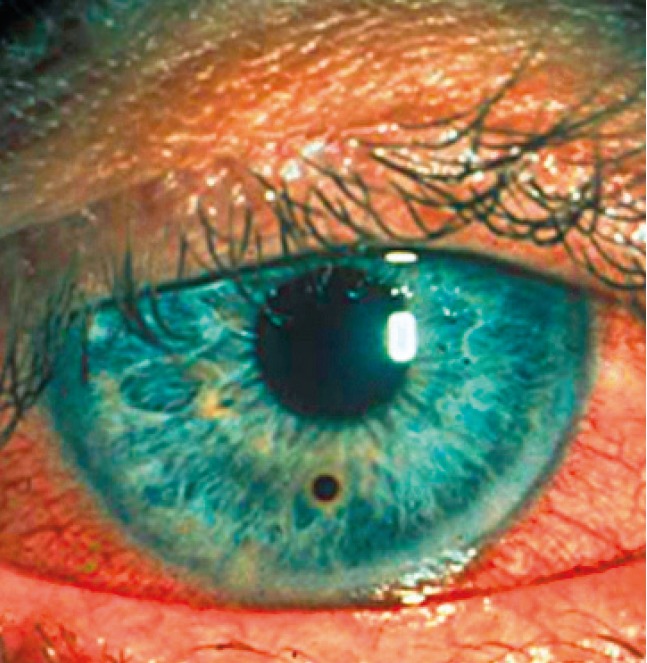	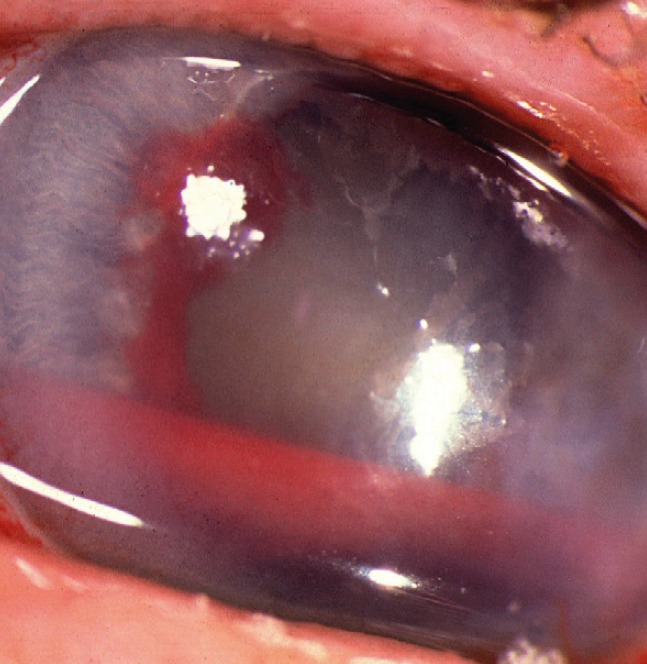	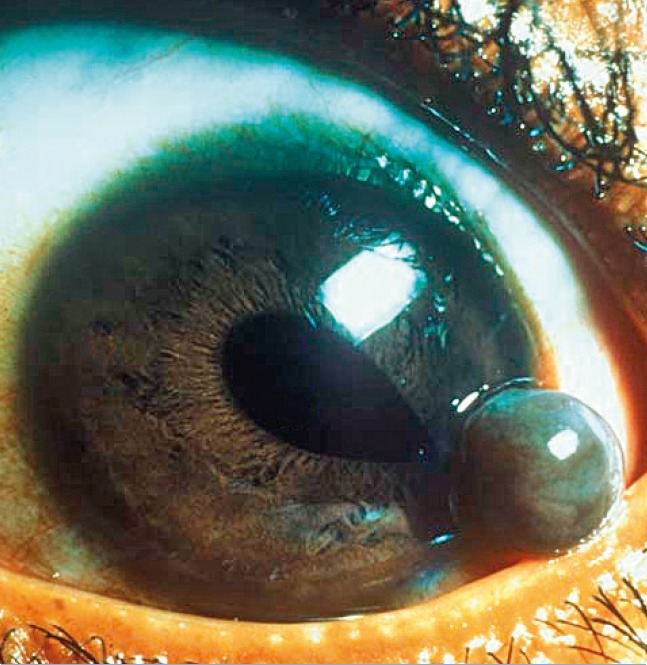	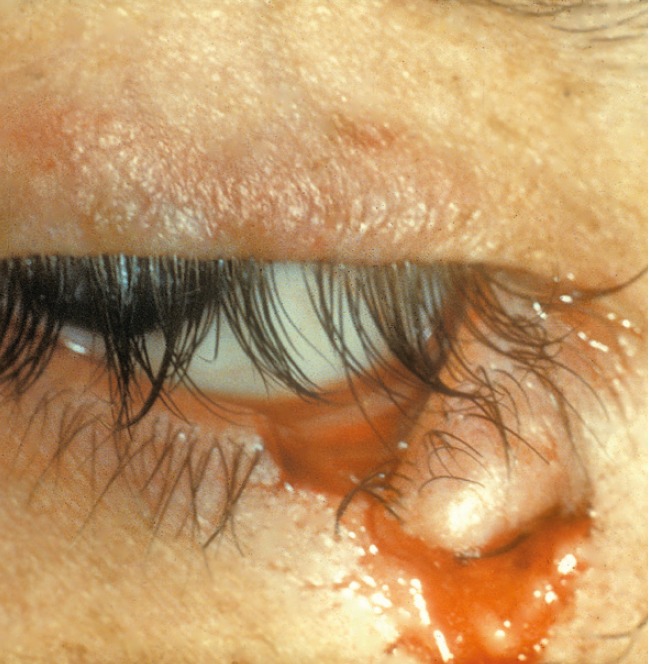	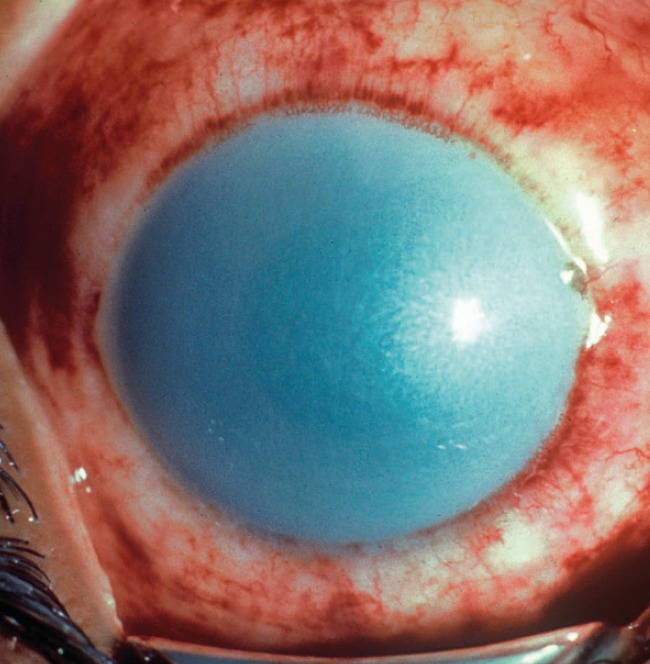
	**Foreign body**	**Blunt injury**	**Penetrating injury**	**Lid laceration**	**Burns**
**Assessment**	**History** Foreign body sensation. Maybe conjunctival, corneal or sub-tarsal (under the upper eyelid)	**History** Injury by blunt object, e.g. fist, stone, etc. Blood in the front of the eye (anterior chamber hyphaema)	**History** Typically by a sharp object, e.g. stick. Perforation of the ‘coat’ of the eye (cornea or sclera)	**History** Laceration of lid margin or canaliculus	**History** Acid, alkali or thermal injury to the eye
	**Vision** Usually normal but can be affected if central cornea is involved	**Vision** Reduced	**Vision** Reduced	**Vision** Normal	**Vision** Reduced
	**Torch exam** Foreign body is seen on conjunctiva or cornea, or under lid	**Torch exam** Blood seen in anterior chamber. Pupil may be dilated	**Torch exam** Cornea may be hazy and pupil maybe distorted with uveal prolapse	**Torch exam** Laceration visible	**Torch exam** Red eye and hazy cornea
**Management**	**1.** Wash any loose foreign body away with clean water. **2. Conjunctival or subtarsal** foreign bodies can be removed with a clean cotton bud. For a **corneal** foreign body, use local anaesthetic first, then try and gently remove it with the corner of a clean piece of paper. **3.** Apply antibiotic eye ointment	**1.** Apply an eye pad to prevent the person from rubbing the eye **2.** Recommend bed rest and offer pain relief. Analgesics must not contain aspirin	**1.** Apply an eye pad. Be very careful not to press on the eye **2.** Give tetanus toxoid 0.5 ml immediately	Give tetanus toxoid 0.5 ml immediately	**1.** Immediately wash the eye with clean water for 5 minutes **2.** Apply antibiotic eye ointment **3.** Offer pain relief
	**Refer if…**	**Refer if…**	**Refer urgently**	**Refer**	**Refer urgently**
**Referral**	Refer if the foreign body cannot be removed	Refer if the person's vision is reduced, there is more bleeding inside the eye or the eye becomes more painful	Refer to an eye unit immediately	Refer to an eye unit to ensure surgery aligns the lid margin	Refer to an eye unit immediately

